# SWI/SNF-associated molecular subtypes reshape tumor microenvironmental features and inform precision therapeutic strategies in bladder cancer

**DOI:** 10.3389/fcimb.2026.1774929

**Published:** 2026-02-18

**Authors:** Tiezheng Qi, Wenqi Yang, Rongxin Liu, Dingshan Deng, Xianghu Liu

**Affiliations:** 1 Department of General Surgery, Xiangya Hospital of Central South University, Changsha, Hunan, China; 2 Xiangya School of Medicine, Central South University, Changsha, Hunan, China; 3 Department of Urology, The First Affiliated Hospital of Guangzhou Medical University, Guangzhou, Guangdong, China; 4 Department of Urology, Hunan Provincial People’s Hospital the First Affiliated Hospital of Hunan Normal University, Changsha, Hunan, China; 5 Department of Emergency Medicine, The Affiliated Changsha Central Hospital, Hengyang Medical School, University of South China, Changsha, Hunan, China

**Keywords:** bladder cancer, immunotherapy, molecular subtyping, SWI/SNF chromatin-remodeling complex, tumor microenvironment

## Abstract

**Background:**

Bladder cancer (BLCA) is a malignant tumor characterized by pronounced molecular and immunological heterogeneity. Despite continuous advances in immunotherapy and targeted treatment, clinical outcomes vary substantially, and effective molecular stratification and predictive biomarkers remain lacking. The SWI/SNF chromatin-remodeling complex regulates chromatin accessibility and gene transcription through ATP-dependent mechanisms and frequently harbors mutations across multiple cancer types. However, whether SWI/SNF-related molecular patterns can be leveraged for clinically meaningful stratification and therapeutic prediction in BLCA remains unclear.

**Methods:**

We integrated the TCGA-BLCA dataset, the IMvigor210 immunotherapy cohort, GEO datasets, and an independently sequenced Xiangya RNA cohort to comprehensively analyze SWI/SNF-related genes. Unsupervised clustering was applied to construct SWI/SNF molecular subtypes, and a quantitative scoring system, SWI_SNF_Score, was established based on differentially expressed and prognostically relevant genes. Using transcriptomic profiling across multiple cohorts and bioinformatics approaches, we assessed the relationships between SWI_SNF_Score, molecular subtypes, clinical outcomes, characteristics of the tumor immune microenvironment, and therapeutic responses, followed by validation in independent cohorts.

**Results:**

Two distinct SWI/SNF-associated molecular subtypes were identified across integrated transcriptomic datasets. Based on differentially expressed genes, we constructed the SWI_SNF_Score to quantitatively represent SWI/SNF-related molecular and immune features in BLCA. A high SWI_SNF_Score was significantly associated with the basal subtype, enhanced stromal and immune activation, frequent RB1/TP53 co-mutations, and unfavorable prognosis. In contrast, a low SWI_SNF_Score displayed luminal differentiation features, enrichment of FGFR3/KDM6A mutations, and favorable survival outcomes. The SWI_SNF_Score accurately predicted classical molecular subtypes, TME phenotypes, and multiple treatment sensitivities and was independently validated in both the Xiangya and IMvigor210 immunotherapy cohorts. Notably, the score exhibited distinct predictive value across different immune phenotypes, enabling more precise stratification of potentially responsive populations.

**Conclusions:**

The SWI_SNF_Score enables quantitative evaluation of molecular and immune heterogeneity in bladder cancer and provides a clinically relevant framework for prognostic stratification and immunotherapy response prediction.

## Introduction

Bladder cancer (BLCA) is the 10th most prevalent malignancy worldwide and includes 3 major clinical types: non–muscle-invasive bladder cancer (NMIBC), muscle-invasive bladder cancer (MIBC), and metastatic bladder cancer. BLCA has high incidence and mortality rates, creating a substantial socioeconomic burden (Tran et al., 2021; Ma et al., 2022; Ascione et al., 2023; Dyrskjøt et al., 2023; Zi et al., 2024). At the individual level, the pronounced heterogeneity of the tumor microenvironment (TME) frequently results in highly variable clinical outcomes, most of which are associated with unfavorable prognosis (Hu et al., 2021a; Liang et al., 2023). Although therapeutic approaches for BLCA have continued to evolve, the overall clinical benefit remains limited, particularly in advanced and metastatic disease (Li et al., 2024). Beyond the long-standing reliance on surgery and chemotherapy, newly developed strategies such as immunotherapy and perioperative drug interventions have gradually entered the therapeutic framework for BLCA (Boorjian et al., 2021; Hu et al., 2022; Hu et al., 2025a; Hu et al., 2025b). However, the lack of robust biomarkers or molecular stratification tools that can accurately predict clinical responses and therapeutic efficacy continues to pose a major challenge in BLCA management (Dyrskjøt et al., 2023; Maas et al., 2023). Therefore, achieving precision oncology in BLCA requires the development of novel molecular stratification strategies and quantitative analytical tools that can guide treatment decisions and clinical practice (Xu et al., 2022).

The SWI/SNF chromatin-remodeling complex is a highly conserved multicomponent protein family composed of 9 to 12 subunits encoded by genes originally identified through two independent genetic screens in Saccharomyces cerevisiae (Wilson and Roberts, 2011). This complex plays a central regulatory role in gene expression. It modulates chromatin compaction and accessibility through an ATP-dependent mechanism and thereby controls transcriptional activity (Schaefer and Hornick, 2021). The discovery of recurrent mutations affecting SWI/SNF subunits across various cancers has established a mechanistic link between chromatin remodeling and tumor suppression. Large-scale cancer genome sequencing studies have revealed a high prevalence of mutations in genes encoding SWI/SNF subunits, and approximately 25% of malignancies exhibit genetic aberrations involving one or more components. These alterations are closely associated with tumor initiation and progression (Mittal and Roberts, 2020). Collectively, these findings underscore the biological and therapeutic relevance of SWI/SNF dysregulation in cancer.

In BLCA, loss of critical SWI/SNF components such as ARID1A induces transcriptional and translational conflict, which impairs the synthesis of pro-proliferative proteins (Jana et al., 2023). This mechanism may partially restrict early tumor progression while exerting wide-ranging effects on BLCA biology and therapeutic response (Malone and Roberts, 2024). Beyond tumor-intrinsic effects, accumulating evidence from other cancer types suggests that SWI/SNF alterations can influence immune-related pathways, including interferon signaling, antigen presentation, and inflammatory responses, thereby reshaping the tumor immune microenvironment. Emerging therapeutic strategies targeting the SWI/SNF complex have demonstrated promising potential in multiple cancer types. For example, depletion of SWI/SNF ATPase subunits such as SMARCA2 or SMARCA4 rapidly suppresses oncogenic enhancer-driven transcriptional programs and inhibits tumor progression (Xiao et al., 2022). In addition, when SMARCB1 is lost, novel molecular regulators such as DCAF5 become essential for cancer cell survival. Pharmacologic inhibition of DCAF5 can restore SWI/SNF complex function and reverse malignant phenotypes (Radko-Juettner et al., 2024). However, the role of SWI/SNF dysregulation in shaping the BLCA tumor microenvironment and antitumor immunity has not been systematically characterized.

Taken together, current evidence suggests a potential but incompletely understood link between SWI/SNF alterations, tumor immune regulation, and therapeutic response in BLCA. To advance research in this field, we systematically evaluated the roles of SWI/SNF-related genes in the BLCA tumor microenvironment and confirmed their essential contributions to multiple steps of the antitumor immune cycle. We then established a quantitative scoring system, referred to as the SWI_SNF_Score, which is designed to assess individual TME states and immune potential. This framework provides mechanistic insight into BLCA biology and supports improved prognostic evaluation and precision-guided therapeutic planning. By addressing this critical knowledge gap, our study aims to provide an integrative framework that links SWI/SNF-driven molecular heterogeneity with immune phenotypes, prognosis, and therapeutic responsiveness in BLCA.

## Methods

### Xiangya transcriptomic cohort

This cohort was independently collected and sequenced by Xiangya Hospital and included 57 bladder cancer (BLCA) samples and 13 normal bladder tissues. The samples were processed to generate mRNA sequencing profiles, and detailed descriptions can be found in our previous studies (Hu et al., 2021a; Sheng et al., 2021; Cai et al., 2023; Li et al., 2023). The Xiangya cohort has been deposited in the Gene Expression Omnibus (GEO) database under accession number GSE1887155 (Liu et al., 2021).

### The cancer genome atlas BLCA cohort

The TCGA-BLCA cohort consists of transcriptomic profiles and clinical annotations from 400 BLCA patients. The dataset was initially downloaded from the UCSC Xena platform. After primary data processing and normalization, three formats of RNA sequencing data were retained, including raw counts, fragments per kilobase per million reads (FPKM), and transcripts per million reads (TPM), all of which reflected protein-coding genes only. Somatic copy number variation (CNV) was analyzed using GISTIC2.0 (Mermel et al., 2011), and tumor mutational burden (TMB) was calculated using VarScan2.

### IMvigor210 immunotherapy cohort

IMvigor210 is a clinical immunotherapy trial that evaluated atezolizumab, a PD-L1–blocking antibody, in BLCA patients who were ineligible for cisplatin-based therapy (Balar et al., 2017). We obtained and curated the integrated RNA sequencing matrix with corresponding clinical annotations from the study by Mariathasan and colleagues (Mariathasan et al., 2018).

### Gene expression omnibus cohorts

GSE48075 is a transcriptomic dataset of BLCA (Choi et al., 2014), whereas GSE32894 represents urinary tract carcinoma (Robertson et al., 2017). Both datasets were retrieved from the GEO database. To reduce batch effects, the two datasets were merged into a single meta-cohort using the “sva” package in R.

### Selection of SWI/SNF-related genes and unsupervised clustering

We comprehensively collected all potential genes related to the SWI/SNF chromatin-remodeling complex. The sources included REACTOME, gene set enrichment analysis (GSEA), Gene Ontology (GO), and PUBMED, and a raw gene list containing 1,653 genes was constructed, which we referred to as the Raw_Gene_Set. All included genes were derived exclusively from studies conducted in humans.

Next, univariate Cox regression was performed on the Raw_Gene_Set, yielding 32 prognostically relevant genes (p< 0.001). These genes were incorporated into the Cluster_Gene_Set and subjected to unsupervised clustering. After systematic evaluation, k = 2 was determined to provide the most stable clustering outcome, generating two SWI/SNF-associated molecular clusters, namely Cluster1 (n = 275) and Cluster2 (n = 125), which were referred to as SWI_SNF_Cluster.

### Construction of the SWI/SNF Scoring System: SWI_SNF_Score

The SWI_SNF_Score was derived from the SWI_SNF_Cluster. Using the “limma” package in R, we identified 919 differentially expressed genes (DEGs) between the two clusters. Univariate Cox regression was again applied to screen for DEGs with prognostic relevance, yielding 285 significant genes. Principal component analysis (PCA) was performed on these 285 genes. The loading vectors of the first principal component (PC1) were used as the coefficient matrix for score calculation. Specifically, the formula is written as: ${\text{SWI\_SNF\_Score}}_{i}=\sum_{k=1}^{285}\times {\text{PC\_}}_{1_{ik}}*{\text{Z}}_{{\text{Exp}}_{ik}}$.

${\text{SWI\_SNF\_Score}}_{\text{i}}$ represents the quantitative SWI_SNF_Score value for the  i−th BLCA patient. For example, *k* denotes the index of a gene, ranked from 1 to 285, representing a given gene *Gene_k*. $PC{\_}_{1}ik$ is the coefficient of *Gene_k* on the first principal component. $Z_{Expik}$ is the Z-score–standardized expression level of Gene_k.

### Classical molecular subtyping of bladder cancer

Molecular classification of BLCA has important diagnostic, prognostic, and therapeutic implications. Based on our previous studies (Hu et al., 2021a; Liu et al., 2021; Hu et al., 2022; Cai et al., 2023; Li et al., 2023; Hu et al., 2025a; Hu et al., 2025b), multiple classification frameworks have been widely used and validated (Sjödahl et al., 2012; Choi et al., 2014; Damrauer et al., 2014; Rebouissou et al., 2014; Robertson et al., 2017; Mo et al., 2018; Kamoun et al., 2020; Woerl et al., 2020; Zeng et al., 2020; Tate et al., 2021; He et al., 2022; Li et al., 2024). In this study, molecular subtyping was performed using RNA sequencing data from publicly available cohorts through the “ConsensusMIBC” and “BLCAsubtyping” packages in R. By integrating our SWI_SNF_Score system, we further evaluated its predictive potential for different molecular subtypes. Predictive accuracy was quantified using receiver operating characteristic (ROC) analysis and area under the curve (AUC).

### Survival analysis, enrichment analysis, and mutational profiling

Kaplan–Meier survival analysis was performed using the “survival” package in R, and visualization was performed using “survminer.” For functional annotation, gene set enrichment at the single-sample level was estimated using the single-sample gene set enrichment analysis (ssGSEA) method. The gene set variation analysis (GSVA) approach was used to score molecular signatures across different cohorts. Gene set enrichment analysis (GSEA) was applied to evaluate the biological functions of DEGs, followed by GO and Kyoto Encyclopedia of Genes and Genomes (KEGG) annotation to identify relevant pathways. Mutational profiling was visualized using the “maftools” package, and mutational differences were evaluated using chi-square tests.

### Immune features of the tumor microenvironment

Immune microenvironment analysis was conducted following methodologies from previous studies (Hu et al., 2021a; Hu et al., 2021b; Cai et al., 2023; Li et al., 2023; Yan et al., 2025). At the molecular level, we examined correlations between SWI_SNF_Score and immune-regulatory factors as well as representative immune cell markers. At the cellular level, tumor-infiltrating immune cells (TIICs) were estimated using MCP-COUNTER and TIMER, and the activation of the cancer immunity cycle was evaluated using the TIP platform. Enrichment analysis was subsequently performed to identify immune-related pathways associated with the SWI_SNF_Score.

### Prediction of therapeutic responses in BLCA

We compared treatment-associated mutational features between high and low SWI_SNF_Score groups. Based on previous literature, chemotherapy-related genes such as TP53, RB1, and ERCC2 were selected (Motterle et al., 2020). For erdafitinib-targeted therapy, FGFR2 and FGFR3 mutations were specifically examined (Burki, 2019; Loriot et al., 2019; Siefker-Radtke et al., 2022; Guercio et al., 2023; Loriot et al., 2023). Additional analyses included radiotherapy-associated markers, targeted therapy signatures, and immunotherapy-relevant pathways.

In immune checkpoint blockade (ICB) analyses, particular attention was given to correlations between SWI_SNF_Score and markers of therapeutic response, including the T cell inflammation score (TIS), epithelial–mesenchymal transition (EMT) features, and TGF-β response signatures.

### Statistical analysis and visualization

All statistical analyses and visualizations were performed in R software (version 4.3.3). A p-value below 0.05 was considered statistically significant. Patients with BLCA were dichotomized according to the median or the optimal cutoff of the SWI_SNF_Score.

For variables that met normal distribution assumptions, the Student’s t test was used for comparison between two groups. For skewed distributions, the Mann–Whitney U test was applied. Categorical variables were compared using chi-square tests. All statistical procedures adhered to biomedical analytical logic, and all hypothesis tests were conducted in a two-sided manner.

### Data availability

#### RNA sequencing data with clinical information

Data are available from the corresponding author upon reasonable request.

The Xiangya RNA sequencing cohort (GSE188715) can be accessed in the GEO database: https://www.ncbi.nlm.nih.gov/geo/query/acc.cgi?acc=GSE188715.

GSE32894: https://www.ncbi.nlm.nih.gov/geo/query/acc.cgi?acc=GSE32894.

GSE48075: https://www.ncbi.nlm.nih.gov/geo/query/acc.cgi?acc=GSE48075.

TCGA-BLCA cohort: https://xenabrowser.net/.

IMvigor210: http://research-pub.gene.com/IMvigor210CoreBiologies/.

#### Gene collection databases

MSigDB: https://www.gseamsigdb.org/gsea/msigdb.

GenAge: https://genomics.senescence.info/genes/index.html.

CellAge: https://genomics.senescence.info/cells/query.php?search=.

Digital Ageing Atlas (DAA): https://ageing-map.org/atlas/results/?sort=name&s=&species%5B%5D=9606&t=tissue.

AgeAnno: https://relab.xidian.edu.cn/AgeAnno/#/.

#### Drug databases

DrugBank: https://go.drugbank.com/.

Kaplan–Meier Analysis in BLCA Patients.

https://kmplot.com/ (Győrffy, 2024).

## Results

### Classification of SWI/SNF clusters based on RNA sequencing and mRNA expression

**Figure 1:**
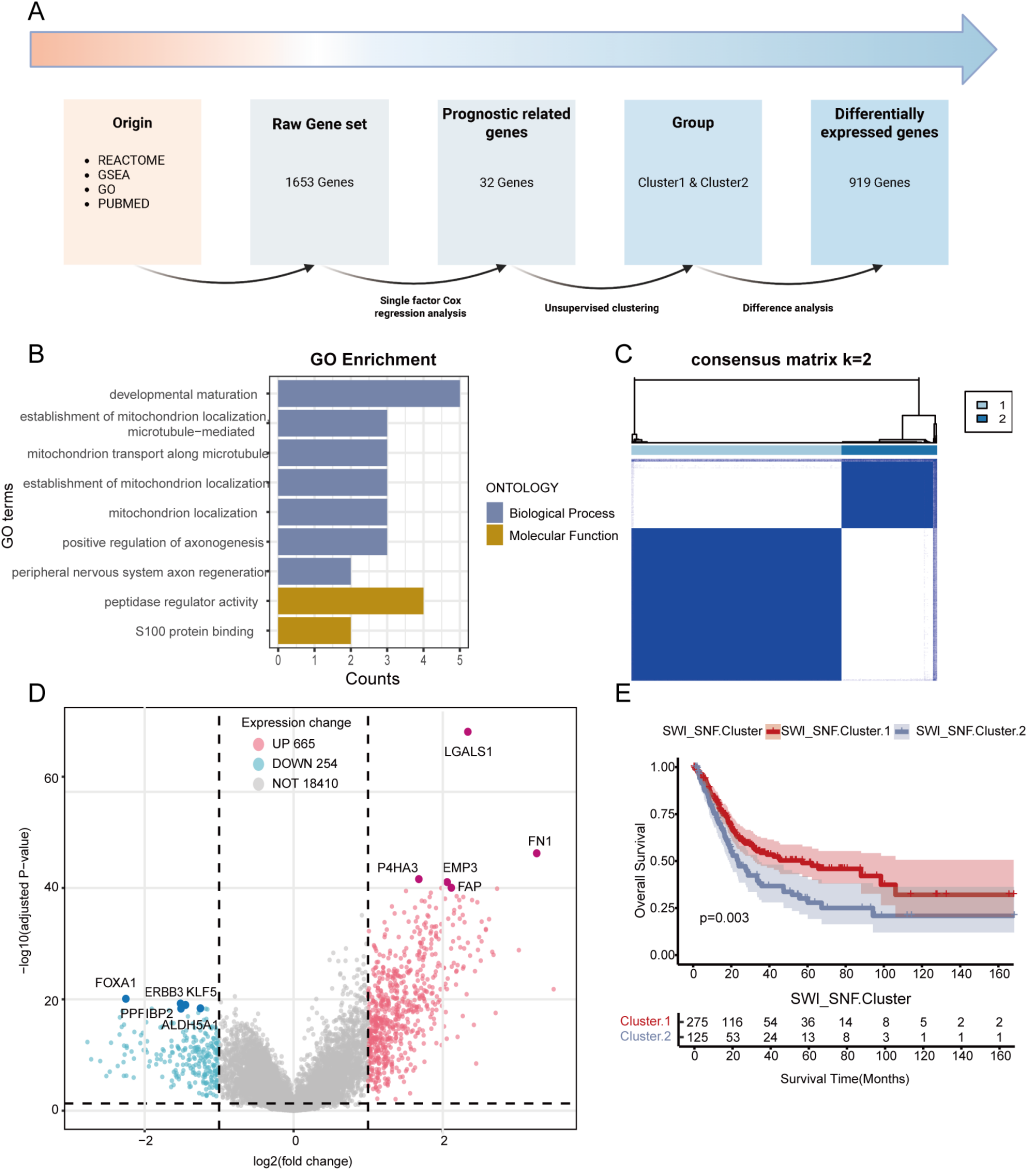
Construction of the SWI/SNF expression–related SWI_SNF_Cluster based on RNA sequencing. **(A)** Overall workflow for gene selection and clustering. **(B)** GO enrichment results of the 32 genes identified by univariate Cox analysis, showing enrichment in major biological processes and molecular functions. **(C)** Unsupervised clustering heatmap of TCGA-BLCA patients using the COX_Gene_Set with k = 2. **(D)** Volcano plot of differentially expressed SWI/SNF-related genes between clusters, showing distributions of significantly upregulated and downregulated genes. **(E)** Kaplan–Meier survival curves comparing patients in SWI_SNF Cluster 1 and Cluster 2.

**Figure 1A** outlines the analytical workflow. As shown in **Figure 1A** and **Supplementary Table S1**, we identified 1,653 feature genes (Raw Gene Set) through systematic screening. Univariate Cox regression analysis prioritized 32 SWI/SNF-associated genes (COX Gene Set), whose functional relevance was corroborated by Gene Ontology (GO) enrichment analysis (**Figure 1B**). Using the 32-gene COX Gene Set, unsupervised consensus clustering of the TCGA-BLCA cohort identified two molecular subgroups: SWI/SNF Cluster 1 (n = 275) and SWI/SNF Cluster 2 (n = 125) (**Figure 1C**; **Supplementary Figures S1A–H**). Differential gene expression patterns between clusters were visualized via heatmap (**Figure 1D**). Kaplan–Meier survival analysis revealed a significant disparity in overall survival between clusters (p< 0.001), with Cluster 2 exhibiting poorer prognosis (**Figure 1E**).

### SWI/SNF gene signature construction, SWI_SNF_Score and functional analysis

**Figure 2:**
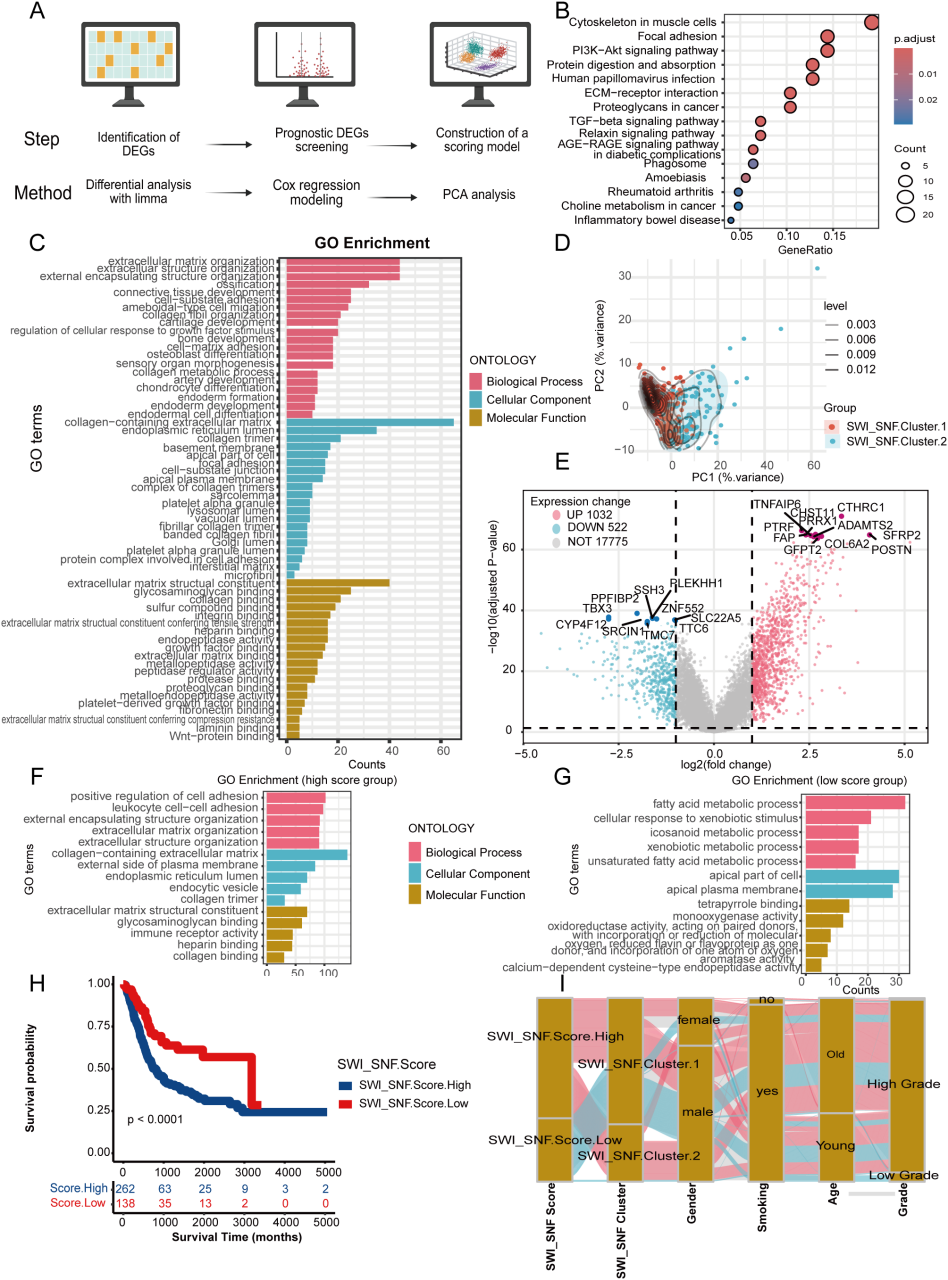
Construction of the SWI_SNF_Score and its association with bladder cancer. **(A)** Workflow for constructing the SWI_SNF_Score. **(B)** KEGG enrichment analysis of differentially expressed genes. **(C)** GO enrichment analysis of differentially expressed genes. **(D)** PCA displaying two SWI/SNF-related subgroups. **(E)** Volcano plot of differentially expressed genes between subgroups. **(F)** GO analysis of genes in the high SWI_SNF_Score group. **(G)** GO analysis of genes in the low SWI_SNF_Score group. **(H)** Survival analysis comparing high and low SWI_SNF_Score groups. **(I)** Sankey diagram showing relationships among the SWI_SNF_Score, molecular subgroups, and clinical characteristics.

**Figure 2A** illustrates the overall workflow for establishing the SWI_SNF_Score. We first identified 919 differentially DEGs between the two SWI/SNF clusters using the “limma” package (**Supplementary Table S4**). Kyoto Encyclopedia of Genes and Genomes (KEGG) enrichment analysis and GO functional annotation were performed on these 285 prognostically significant DEGs, revealing their major biological functions (**Figures 2B, C**). These DEGs were predominantly enriched in tumor-associated pathways and extracellular matrix (ECM) regulatory processes. KEGG analysis indicated significant enrichment in the PI3K–Akt signaling pathway, TGF-beta signaling pathway, and ECM–receptor interaction.

At the biological process (BP) level, the DEGs were enriched in ECM organization, collagen metabolism, and cell–matrix adhesion. At the cellular component (CC) level, the genes were mainly located in ECM structures, collagen-associated regions, and basement membranes. At the molecular function (MF) level, the genes were enriched in collagen binding, integrin binding, and metallopeptidase activity. These results suggest that the DEGs may regulate ECM remodeling and the tumor microenvironment, thereby affecting BLCA development, molecular progression, and patient prognosis.

We then performed PCA on these DEGs to construct the SWI_SNF_Score (**Figure 2D**). Genes upregulated in the high-score group were primarily stromal and ECM-associated markers, including CTHRC1, POSTN, FAP, COL6A2, and ADAMTS2, indicating stromal activation and ECM remodeling (**Figure 2E**). Overall, the SWI_SNF_Score distinguished stromal differentiation characteristics, suggesting potential clinical implications for patient stratification and prognosis assessment.

Functional enrichment analyses revealed pronounced differences between high and low SWI_SNF_Score groups. Genes in the high-score group were enriched in pathways related to cell adhesion, immune regulation, and ECM organization, whereas genes in the low-score group were enriched in metabolic programs such as fatty acid metabolism, redox processes, and xenobiotic metabolism (**Figures 2F, G**). Kaplan–Meier survival analysis confirmed a significant difference in patient prognosis, with the high-score group showing poorer survival outcomes (**Figure 2H**). A Sankey diagram demonstrated the correspondence between SWI_SNF_Score, molecular clusters, gender, and age (**Figure 2I**).

### SWI_SNF_Score in molecular subtypes, therapeutic strategies and mutational patterns

**Figure 3:**
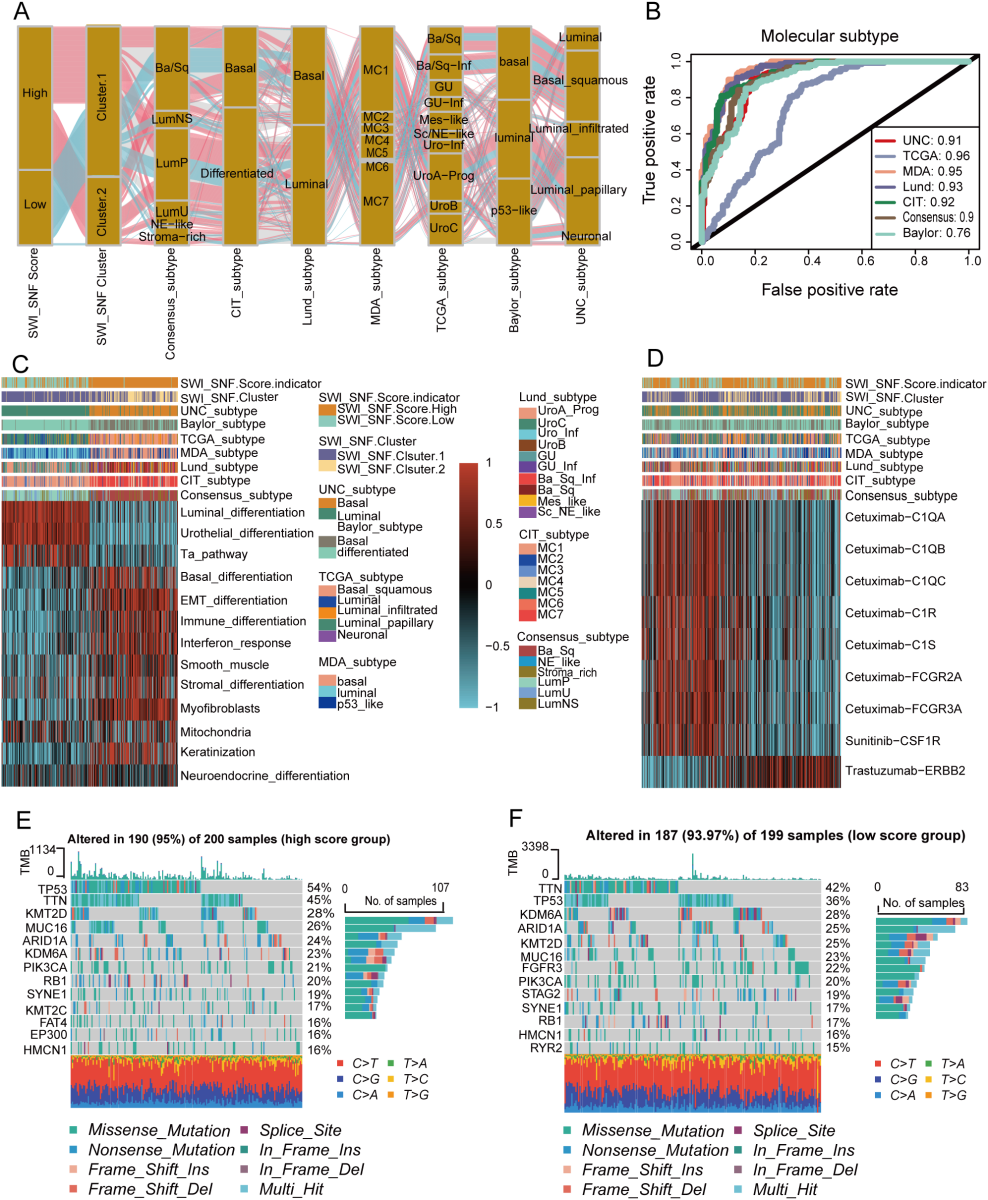
Molecular subtype associations and treatment relevance of the SWI_SNF_Score in bladder cancer. **(A)** Associations of the SWI_SNF_Score and SWI/SNF clusters with classical molecular subtypes of bladder cancer. **(B)** ROC analysis evaluating the predictive performance of the SWI_SNF_Score for classical molecular subtypes. **(C)** Pathway enrichment heatmap of SWI_SNF_Score groups and SWI/SNF clusters annotated with molecular subtype information. **(D)** Treatment sensitivity analysis of high and low SWI_SNF_Score groups. **(E)** Mutation landscape of the high SWI_SNF_Score group. **(F)** Mutation landscape of the low SWI_SNF_Score group.

The SWI_SNF_Score demonstrated clear stratification across multiple molecular classification systems. Samples with high scores were predominantly enriched in basal differentiation subtypes and exhibited EMT and immune activation features. In contrast, samples with low scores showed luminal differentiation, urothelial differentiation, and Ta pathway characteristics (**Figure 3A**). ROC analysis confirmed the discriminative performance of the SWI_SNF_Score across Baylor, UNC, MDA, and TCGA systems, AUC values greater than 0.8, indicating strong prediction accuracy for molecular subtypes (**Figure 3B**). Consistent trends were observed in pathway enrichment heatmaps. The high-score group showed elevated expression in basal differentiation, EMT differentiation, and immune differentiation, while the low-score group was characterized by luminal differentiation, urothelial differentiation, and Ta pathway signatures (**Figure 3C**). Previous studies on molecular stratification have shown similar patterns, where the high-score group corresponds to a more aggressive tumor phenotype and the low-score group aligns with favorable prognostic tendencies (Dyrskjøt et al., 2023).

In therapeutic response analyses, the two SWI_SNF_Score groups displayed distinct patterns (**Figure 3D**). The high-score group exhibited greater activity in EGFR signaling and immune-related signatures, suggesting potential sensitivity to EGFR-targeted therapy, immune checkpoint blockade, and radiotherapy. Conversely, the low-score group showed higher enrichment in Sunitinib–CSF1R and Trastuzumab–ERBB2 signatures, indicating possible suitability for CSF1R-targeting or HER2-targeting therapeutic strategies.

Mutational landscape analysis revealed a high mutational burden in both groups, with an overall mutation rate of 93.97 percent. TP53 was frequently mutated in both score groups. However, the accompanying mutation patterns differed substantially. The high-score group exhibited more frequent RB1 co-mutations, which matched basal and aggressive phenotypes. The low-score group was enriched with FGFR3 and KDM6A mutations, which were biologically consistent with luminal molecular features (**Figures 3E, F**). These findings demonstrate that the SWI_SNF_Score not only differentiates molecular subtypes, but also reflects differences in treatment susceptibility and mutational architecture, thereby offering potential implications for individualized treatment in BLCA.

### SWI_SNF_Score as a distinguishing factor of tumor immune microenvironment states

**Figure 4:**
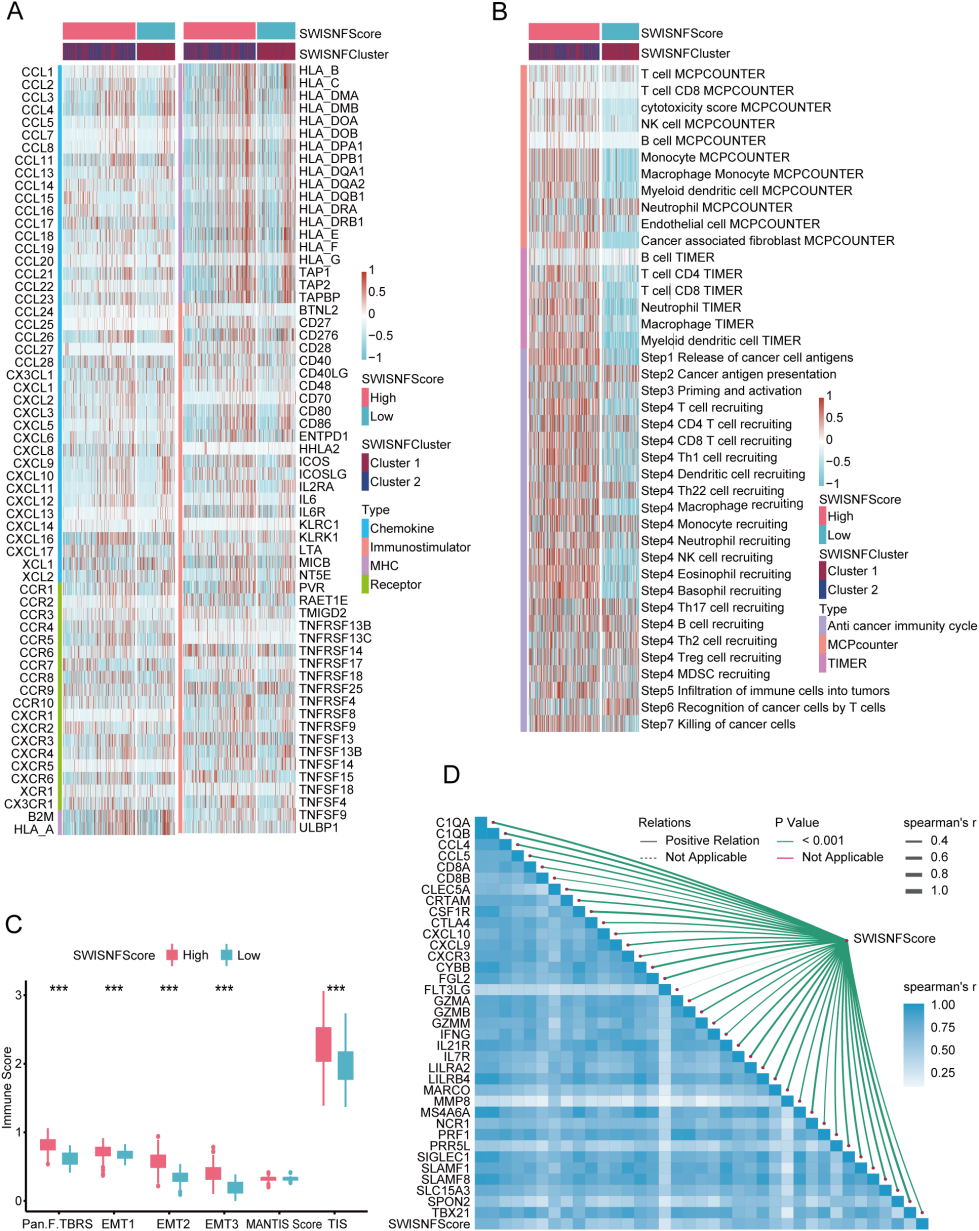
The SWI-SNF-Score effectively distinguishes the immune status of the tumor immune microenvironment. **(A)** Expression of immune-regulatory factors in SWI_SNF_Score groups and SWI_SNF clusters. **(B)** The levels of immune infiltration and anti-cancer immune cycle prediction in the SWI-SNF-Score and SWI-SNF-Cluster groups. **(C)** Differences in immune scores between high and low SWI_SNF_Score groups. **(D)** Correlations between the SWI_SNF_Score and effector genes of immune cells.

The high SWI_SNF_Score group and the SWI_SNF_Cluster2 group exhibited elevated expression of immune-regulatory factors (**Figure 4A**). Consistent with this observation, the high SWI_SNF_Score group and the SWI_SNF_Cluster2 group showed significantly higher levels of immune cell infiltration (**Figure 4B**). As expected, most steps of the antitumor immune cycle were more activated in the high SWI_SNF_Score group and in the SWI_SNF_Cluster2 group (**Figure 4B**). The immune score was significantly higher in the high SWI_SNF_Score group compared with the low-score group (**Figure 4C**). All effector signatures showed strong correlations with the SWI_SNF_Score (**Figure 4D**), indicating enhanced immune infiltration in the high-score group.

### SWI_SNF_Score as a distinguishing factor of immunotherapy responses

**Figure 5:**
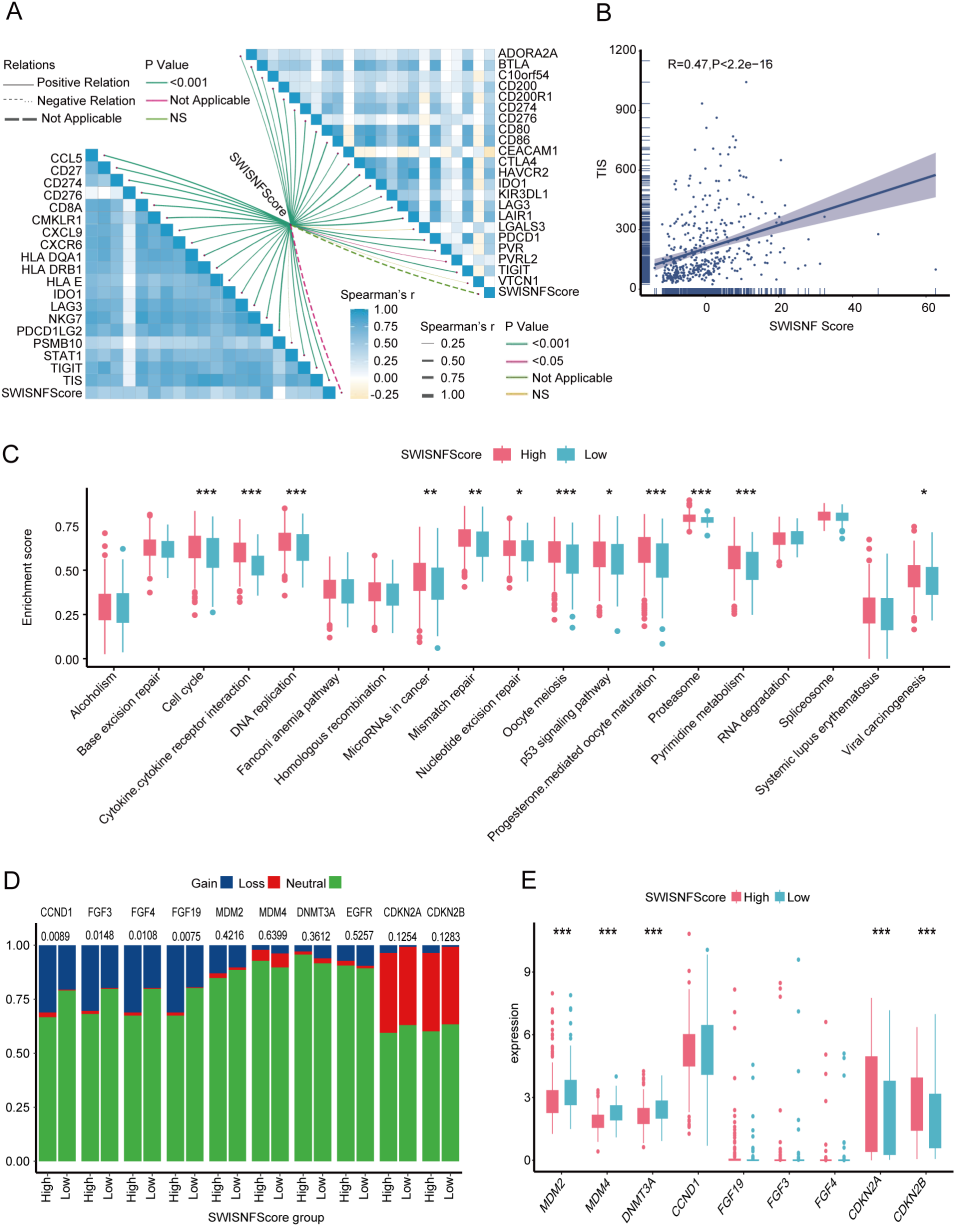
The SWI_SNF_Score reflects immunotherapy effectiveness. **(A)** Correlations between the SWI_SNF_Score, TIS effector genes, and immune checkpoint molecules. **(B)** Linear correlation between the SWI_SNF_Score and the T cell inflammation score (TIS). **(C)** Differences in enrichment scores of immune checkpoint inhibitor–related pathways between high and low SWI_SNF_Score groups. **(D)** Mutational differences in immunotherapy progression–related genes between the two SWI_SNF_Score groups. **(E)** Expression differences of immunotherapy progression–related genes between the two SWI_SNF_Score groups. * P < 0.05, ** P < 0.01, and *** P < 0.001.

Compared with the low-score group, the high SWI_SNF_Score group exhibited a stronger T cell inflammation score (TIS), indicating more pronounced immune cell infiltration. A linear relationship between the SWI_SNF_Score and TIS was confirmed, with a slope of 0.47, highlighting a strong correlation between the two variables (p< 0.001, **Figure 5B**). The SWI_SNF_Score was significantly associated with most key genes used to construct the TIS, except PSMB10, which showed no significant correlation (p > 0.05, **Figure 5A**). The SWI_SNF_Score also showed positive correlations with the majority of immune checkpoint molecules, although LGALS3 and VTCN1 did not exhibit statistically significant associations (p > 0.05, **Figure 5A**).

Pathways associated with immune checkpoint inhibitor (ICI) response were significantly upregulated in the high SWI_SNF_Score group (**Figure 5C**). Interestingly, CDKN2A and CDKN2B are genes negatively associated with immune hyper progression (Giusti et al., 2019), and their expression is also significantly enhanced in the high SWI-SNF-Score group (**Figures 5D, E**). Taken together, the high SWI_SNF_Score group is associated with enhanced immune infiltration, indicating the potential benefits of immunotherapy, but it is also associated with potential risks of immune hyper progression and adverse outcomes—highlighting its significant role in predicting the clinical efficacy of ICI.

### Validation of SWI_SNF_Score in the Xiangya cohort

**Figure 6:**
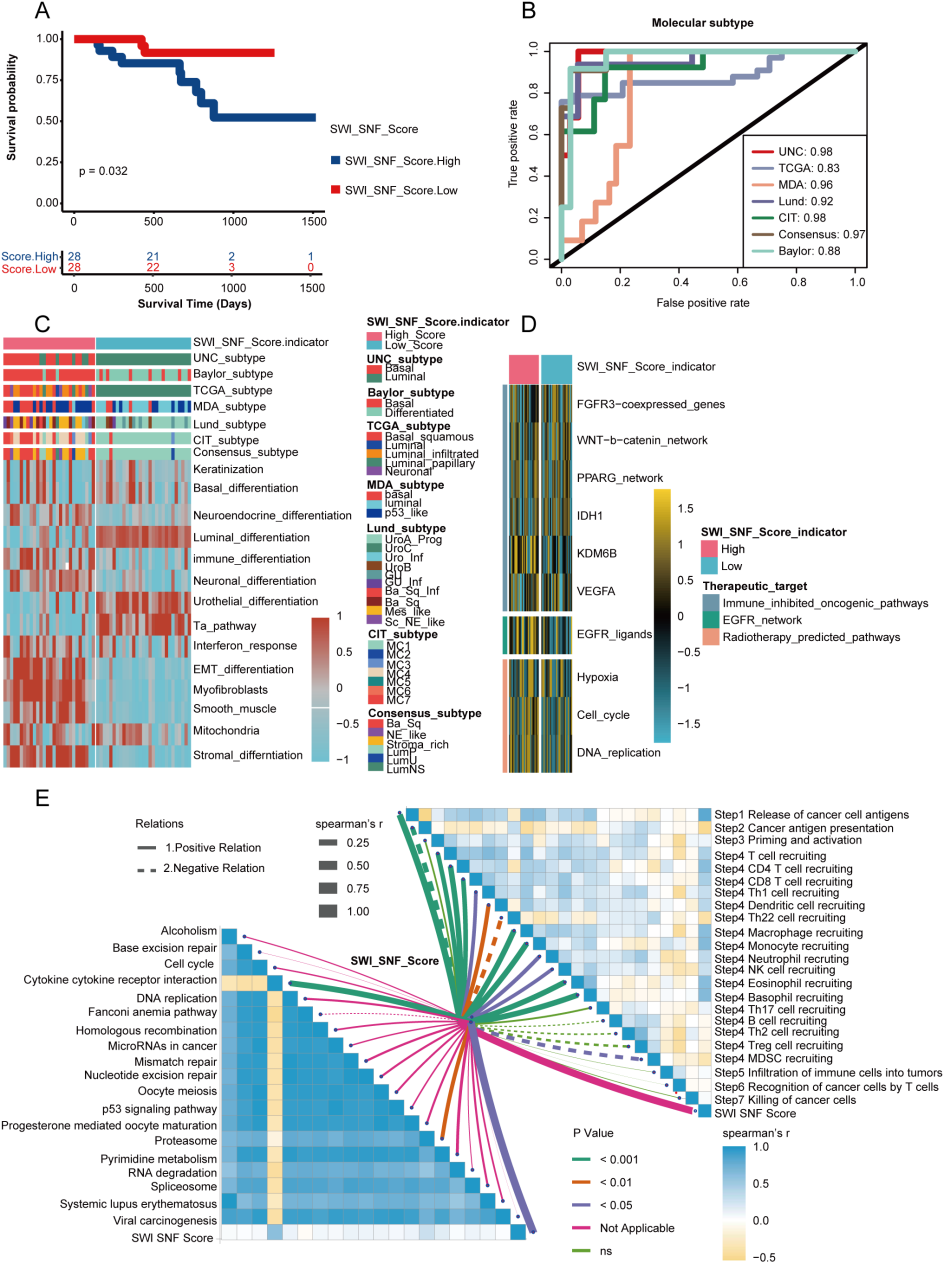
Validation of the SWI_SNF_Score in the Xiangya cohort. **(A)** Survival analysis of high and low SWI_SNF_Score groups. **(B)** ROC analysis showing the predictive ability of the SWI_SNF_Score for molecular subtypes. **(C)** Heatmap showing associations between the SWI_SNF_Score and classical molecular subtypes. **(D)** Pathway enrichment differences between high and low SWI_SNF_Score groups. **(E)** Correlations between the SWI_SNF_Score and steps of the cancer immunity cycle, as well as correlations with signaling pathways.

In the Xiangya cohort, the SWI_SNF_Score exhibited significant associations with the molecular characteristics and clinical outcomes of BLCA. Kaplan–Meier survival analysis showed that patients with high SWI_SNF_Score values had poorer prognosis (p = 0.032, **Figure 6A**). The SWI_SNF_Score demonstrated strong discriminative performance for predicting classical molecular subtypes, with the area under the ROC curve ranging from 0.84 to 0.98 (**Figure 6B**).

Molecular subtype analysis further revealed that the low-score group was enriched for luminal differentiation, urothelial differentiation, and Ta pathway characteristics. In contrast, the high-score group presented stromal-associated features, including fibroblast activation and EMT differentiation (**Figures 6C, D**).

Immune-related analyses indicated that the high SWI_SNF_Score was consistent with an inflammatory tumor microenvironment. The score was significantly correlated with multiple steps of the cancer immunity cycle, including T cell recruitment and natural killer cell activation. Additionally, the high SWI_SNF_Score was closely associated with pathways involving DNA damage repair, cell cycle regulation, and virus-associated carcinogenesis (**Figure 6E**).

Importantly, the SWI_SNF_Score showed strong positive correlations with multiple immune checkpoint molecules, including PDCD1 (PD-1), CD274 (PD-L1), CTLA4, and TIGIT. The score was also highly consistent with the expression of a wide range of immune-related genes (**Supplementary Figure S2A**). Collectively, these results demonstrate that the SWI_SNF_Score not only reflects molecular subtype and prognosis in BLCA, but also reveals immune microenvironment characteristics and pathway features, providing potential value for guiding future clinical decision-making.

### Validation of SWI_SNF_Score in the IMvigor210 cohort

**Figure 7:**
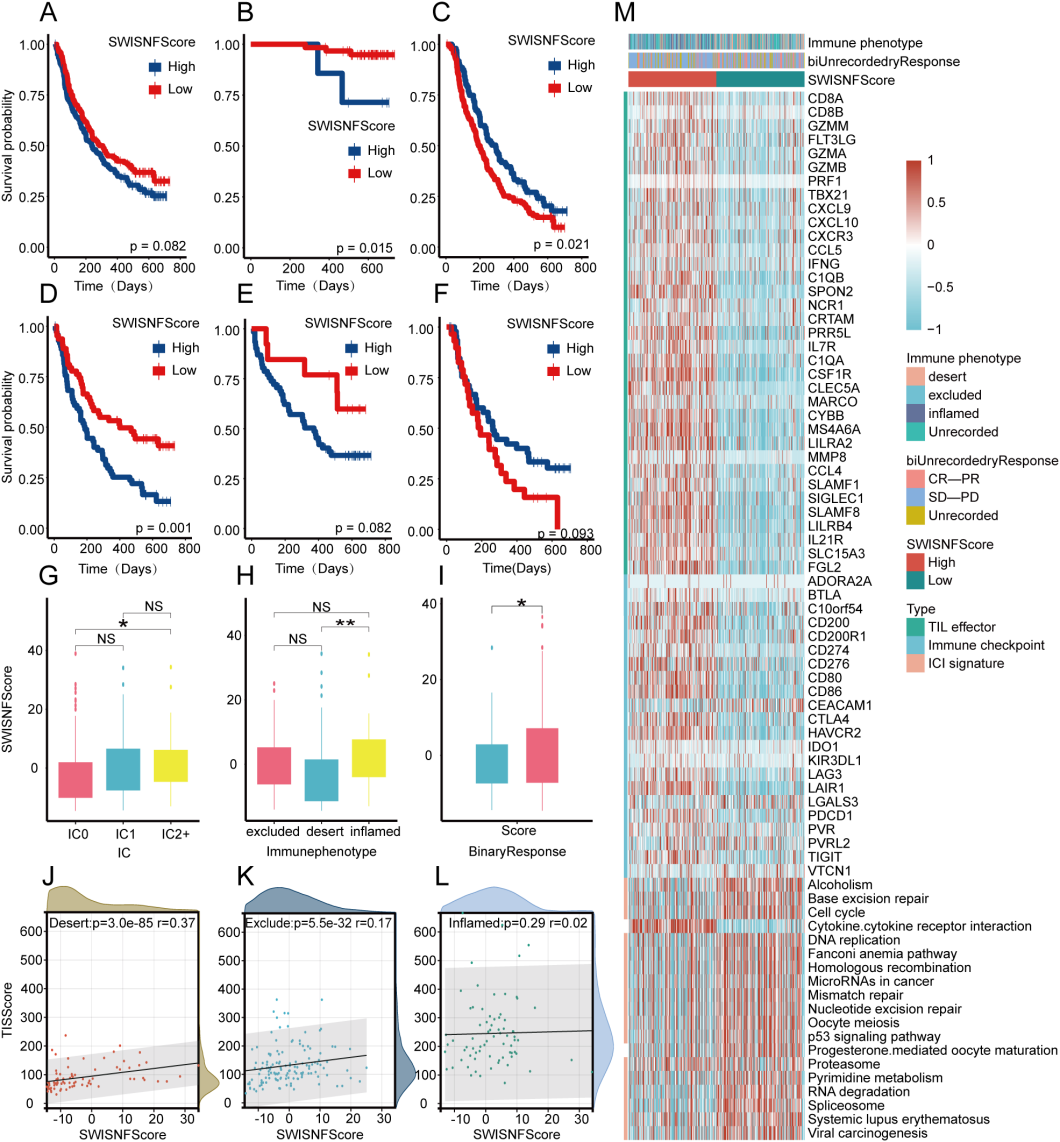
Validation of the SWI_SNF_Score in the IMvigor210 cohort. **(A)** Overall survival differences between SWI_SNF_Score groups. **(B)** Survival differences between SWI_SNF_Score groups in the CR/PR subgroup. **(C)** Survival differences between SWI_SNF_Score groups in the SD/PD subgroup. **(D)** Survival analysis in the excluded immune phenotype. **(E)** Survival analysis in the inflamed immune phenotype. **(F)** Survival analysis in the desert immune phenotype. **(G)** Differences in the SWI_SNF_Score across immune cell subsets. **(H)** Differences in the SWI_SNF_Score across immune phenotypes. **(I)** Differences in the SWI_SNF_Score between binary response groups. **(J)** Linear fitting curve of the relationship between the SWI_SNF_Score and TIS in the desert phenotype. **(K)** Linear fitting curve of the relationship between the SWI_SNF_Score and TIS in the excluded phenotype. **(L)** Linear fitting curve of the relationship between the SWI_SNF_Score and TIS in the inflamed phenotype. **(M)** Expression of TILs, immune checkpoints, and immune checkpoint inhibitor signatures across three immune phenotypes, two SWI/SNF clusters, and SWI_SNF_Score groups. *P < 0.05, ** P < 0.01, and NS, Not Significant.

In the immunotherapy cohort (IMvigor210), the SWI_SNF_Score underwent additional validation. In the overall patient population, there was no significant survival difference between high and low SWI_SNF_Score groups (**Figure 7A**). However, more nuanced findings emerged when patients were further stratified based on treatment response. In the complete response and partial response (CR/PR) subgroup, patients with low SWI_SNF_Score values exhibited more favorable outcomes (**Figure 7B**). In contrast, in the stable disease and progressive disease (SD/PD) subgroup, patients with high SWI_SNF_Score values showed superior outcomes (**Figure 7C**).

Considering the reshaping effect of immunotherapy within the tumor microenvironment, patients in the IMvigor210 cohort were categorized into three immune phenotypes, namely the desert phenotype, the excluded phenotype, and the inflamed phenotype. Regression analysis showed a positive linear correlation between the SWI_SNF_Score and TIS in both the desert and excluded phenotypes, indicating potential predictive utility of the SWI_SNF_Score in these immune contexts (**Figures 7J–L**).

In the excluded phenotype and inflamed phenotype, patients with high SWI_SNF_Score values consistently exhibited poorer prognosis (**Figures 7D, E**). In contrast, in the desert phenotype, patients with high SWI_SNF_Score values unexpectedly demonstrated better outcomes (**Figure 7F**).

We presented the expression profiles of tumor-infiltrating lymphocyte (TIL) effectors, immune checkpoints (IC) markers, and ICI, and observed that TIL and IC markers were upregulated in the high SWI_SNF_Score group across all immune phenotypes, while ICI showed a more significant upregulation in the low SWI_SNF_Score group across all immune phenotypes (**Figure 7M**). Both TIL and ICI signatures demonstrated strong correlations with the SWI_SNF_Score (**Supplementary Figure S3A**). Significant differences in SWI_SNF_Score were also observed across immune cell subsets, immune phenotypes, and binary response patterns (**Figures 7G–I**).

By integrating the SWI_SNF_Score with the three immune phenotypes, clinicians may more effectively select treatment strategies and assess prognosis for patients receiving immunotherapy.

## Discussion

In the field of urologic oncology, regarding the research on the molecular mechanisms of malignant tumors such as BLCA, the roles of mutations in classic oncogenes and tumor suppressor genes (e.g., MYC, RAS, and TP53) in cancer initiation and progression have been intensively studied for decades. A relatively mature system has been established for the related mechanisms as well as their clinical translation and application. By contrast, the discovery of widespread mutations in SWI/SNF complex genes in cancer is relatively recent, emerging only within the past decade. Therefore, our understanding of the interactions between the SWI/SNF complex and BLCA, as well as their associated therapeutic implications, remains at an early developmental stage. In clinical practice, current therapeutic strategies for BLCA are often limited by the intrinsic heterogeneity of tumors and fail to fully meet individualized treatment needs for all patients. These challenges highlight the urgency of further exploring SWI/SNF-related mechanisms in BLCA. In this context, our study introduced a SWI/SNF-based molecular stratification framework and further developed a continuous quantitative index, the SWI_SNF_Score. Compared with traditional discrete subtype classifications, this scoring system enables refined, individualized characterization of BLCA heterogeneity along a continuous spectrum, thereby facilitating more precise prognostic assessment and therapeutic stratification.

Notably, the SWI_SNF_Score is not intended to replace existing clinical biomarkers, but rather to complement them by capturing epigenetic and immune-related dimensions that are not fully reflected by conventional indicators such as PD-L1 expression, tumor mutational burden (TMB), or FGFR mutation status. This complementary potential suggests that integrating the SWI_SNF_Score with established biomarkers may further improve the accuracy of immunotherapy response prediction and patient selection.

Validation across multiple cohorts confirmed the stability and utility of the SWI_SNF_Score under diverse clinical backgrounds, particularly in predicting survival outcomes with high accuracy. In the TCGA-BLCA cohort and the Xiangya cohort (both excluding patients who received immunotherapy), the low SWI_SNF_Score group was consistently associated with better prognosis. In contrast, among immunotherapy recipients in the IMvigor210 cohort, the SWI_SNF_Score tailored prognostic prediction according to the specific immune microenvironment. Notably, patients in the high SWI_SNF_Score group with the desert immune phenotype exhibited more favorable prognostic outcomes, which differed from the results observed in other subgroups and the TCGA cohort. These findings underscore the influence of immunotherapy-induced spatiotemporal heterogeneity on clinical outcomes and highlight the need to further investigate the relationship between the SWI_SNF_Score and the tumor microenvironment. Interestingly, this study revealed an apparently paradoxical pattern in which the high SWI_SNF_Score group displayed features of immune activation yet was associated with unfavorable prognosis in non-immunotherapy settings. A plausible explanation for this phenomenon is that immune infiltration in the high-score group may be dominated by functionally suppressive immune populations, such as exhausted CD8^+^ T cells, regulatory T cells, and immunosuppressive myeloid cells, rather than by effective cytotoxic immune responses. Previous studies have shown that epigenetic dysregulation, including alterations in chromatin remodeling complexes, can profoundly affect immune cell differentiation, exhaustion status, and cytokine signaling, ultimately leading to an “inflamed but immunosuppressed” tumor microenvironment (Binnewies et al., 2018; Hu et al., 2021a; Schenkel and Pauken, 2023; Hu et al., 2025a; Hu et al., 2025b).

From this perspective, the SWI_SNF_Score may reflect not only the quantity but also the functional quality of immune infiltration within the TME. In non-immunotherapy cohorts, the SWI_SNF_Score demonstrated positive associations with numerous immune markers, immune cell populations, and antitumor immune pathways within the tumor microenvironment. In cohorts excluding immunotherapy, the SWI-SNF-Score correlates with numerous immune markers within the TME in a complex manner. The high SWI_SNF_Score group indicates that the TME is enriched with immune modulators, key immune cells, and pathways conducive to antitumor activity. These findings further suggest that patients with high SWI_SNF_Score may benefit from combination therapeutic strategies, such as immune checkpoint blockade combined with targeted therapies or epigenetic modulators, to overcome immune dysfunction and enhance antitumor efficacy.

Despite the compelling findings generated by this study, several limitations should be acknowledged. First, the current work relies primarily on bioinformatic analyses and transcriptomic datasets and lacks experimental validation. Future studies should incorporate *in vitro* experiments and preclinical models to validate the biological mechanisms inferred by our analyses. Second, our analyses have not yet integrated metabolomic, proteomic, or other multi-omics data. Incorporation of additional omics layers would enable more comprehensive elucidation of the molecular mechanisms underlying SWI/SNF-related tumor biology and would strengthen the robustness of our conclusions. In addition, the current SWI_SNF_Score cutoff value was derived from retrospective cohorts and may be influenced by cohort-specific characteristics. Future multicenter, prospective studies are warranted to optimize and validate dynamic cutoff thresholds, enabling broader clinical generalizability.

Methodologically, this study conducted Kaplan-Meier survival analysis based on the TCGA-BLCA cohort, determined the optimal cutoff value of the SWI_SNF_Score by combining ROC curves with the Youden index, and validated it in the Xiangya cohort and the IMvigor210 cohort. However, the selection of the cutoff value may be affected by sample characteristics and event distribution, leading to a certain risk of overfitting. With the continuous accumulation of larger-scale, multicenter, and more heterogeneous clinical data, the current cutoff value may need dynamic adjustment to ensure its generalizability and accuracy in a broader population.

Finally, although the SWI_SNF_Score provides strong preclinical evidence, its translation into real-world clinical practice requires additional validation. Prospective, multicenter, randomized controlled trials are necessary to systematically assess its prognostic value and therapeutic decision-making utility. Beyond its role as a static prognostic biomarker, the SWI_SNF_Score may also hold potential as a dynamic monitoring indicator to evaluate treatment response or disease evolution over time. The development of standardized, reproducible, and clinically feasible assays for SWI_SNF_Score measurement will be essential to facilitate its integration into routine clinical workflows.

## Conclusions

The SWI_SNF_Score effectively characterizes the molecular heterogeneity and immune features of bladder cancer. It demonstrates potential value in prognostic assessment and in predicting therapeutic responses. This scoring system provides a new framework for individualized molecular classification and precision treatment in BLCA.

## Data Availability

The datasets presented in this study can be found in online repositories. The names of the repository/repositories and accession number(s) can be found in the article/**Supplementary Material**.

## Supplementary material

Supplementary Figure 1 Consensus clustering analysis of bladder cancer samples based on SWI/SNF-related gene expression. **(A)** Legend of the consensus matrix. **(B–E)** Consensus matrices for k = 2 to 5. **(F)** Tracking plot of clustering stability for k = 2 to 5. **(G)** CDF curves of consensus clustering. **(H)** Relative changes in the area under the CDF curve (Delta area) for different k values.

Supplementary Figure 2 **(A)** Correlations between the SWI_SNF_Score and immune checkpoint molecules, and correlations between the SWI_SNF_Score and immune cell characteristics.

Supplementary Figure 3 **(A)** Correlation between the SWI_SNF_Score and effector genes of tumor-infiltrating lymphocytes (TILs).

Supplementary Table 1 Raw_Gene_Set. Specific genes contained in the Raw_Gene_Set.

Supplementary Table 2 COX_Gene_Set. Gene set included in Cox regression analysis.

Supplementary Table 3 Gene sets used for unsupervised clustering analysis.

Supplementary Table 4 Principal component analysis results used for score construction.
