# Supplementary figures and images for: SWI/SNF-associated molecular subtypes reshape tumor microenvironmental features and inform precision therapeutic strategies in bladder cancer
Source: Front Cell Infect Microbiol. 2026 Feb 18;16:1774929. doi: 10.3389/fcimb.2026.1774929 (PMC12957257; doi:10.3389/fcimb.2026.1774929)

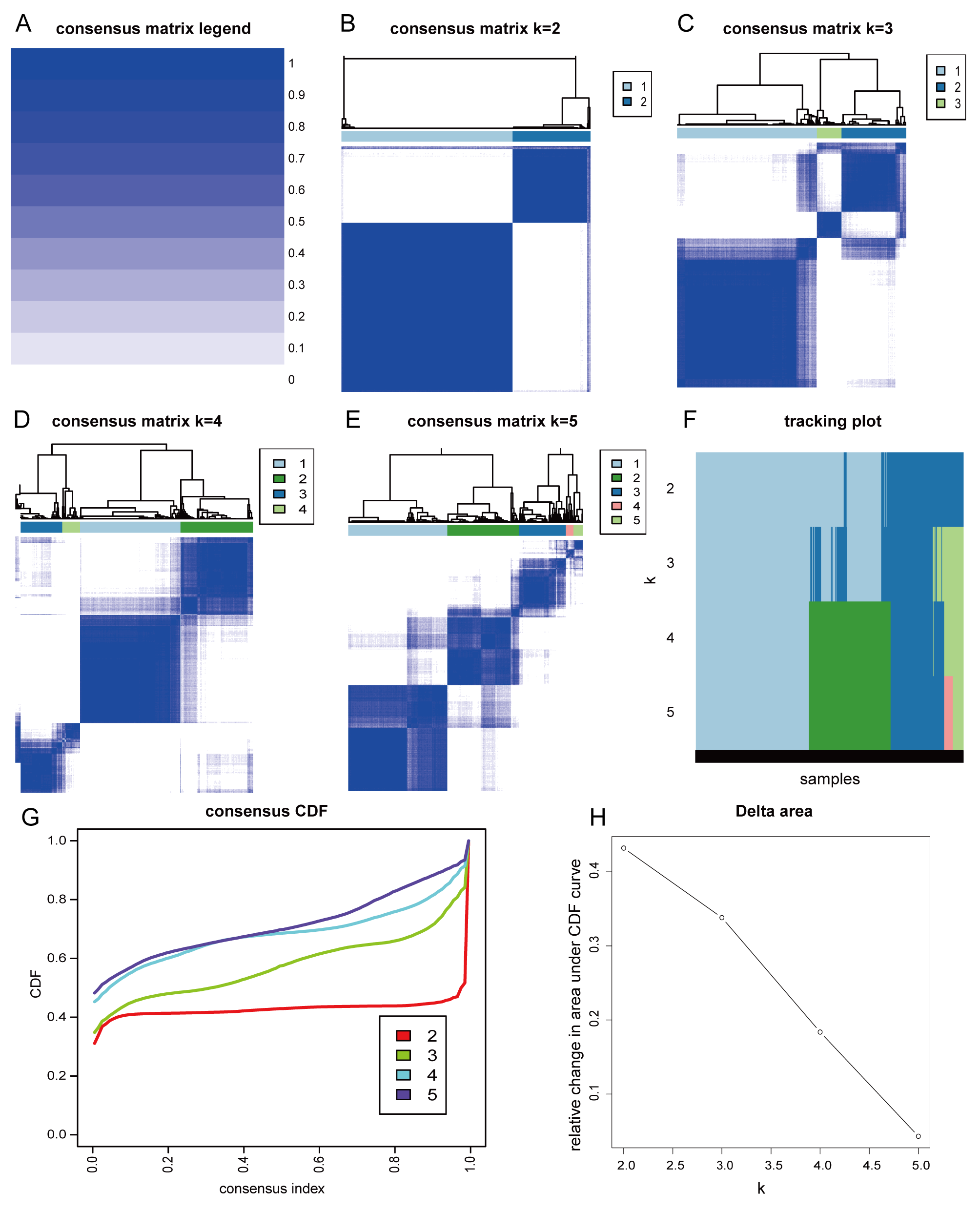

Supplement: Supplementary Figure 1 — Consensus clustering analysis of bladder cancer samples based on SWI/SNF-related gene expression. (A) Legend of the consensus matrix. (B–E) Consensus matrices for k = 2 to 5. (F) Tracking plot of clustering stability for k = 2 to 5. (G) CDF curves of consensus clustering. (H) Relative changes in the area under the CDF curve (Delta area) for different k values. [file Image1.tif]

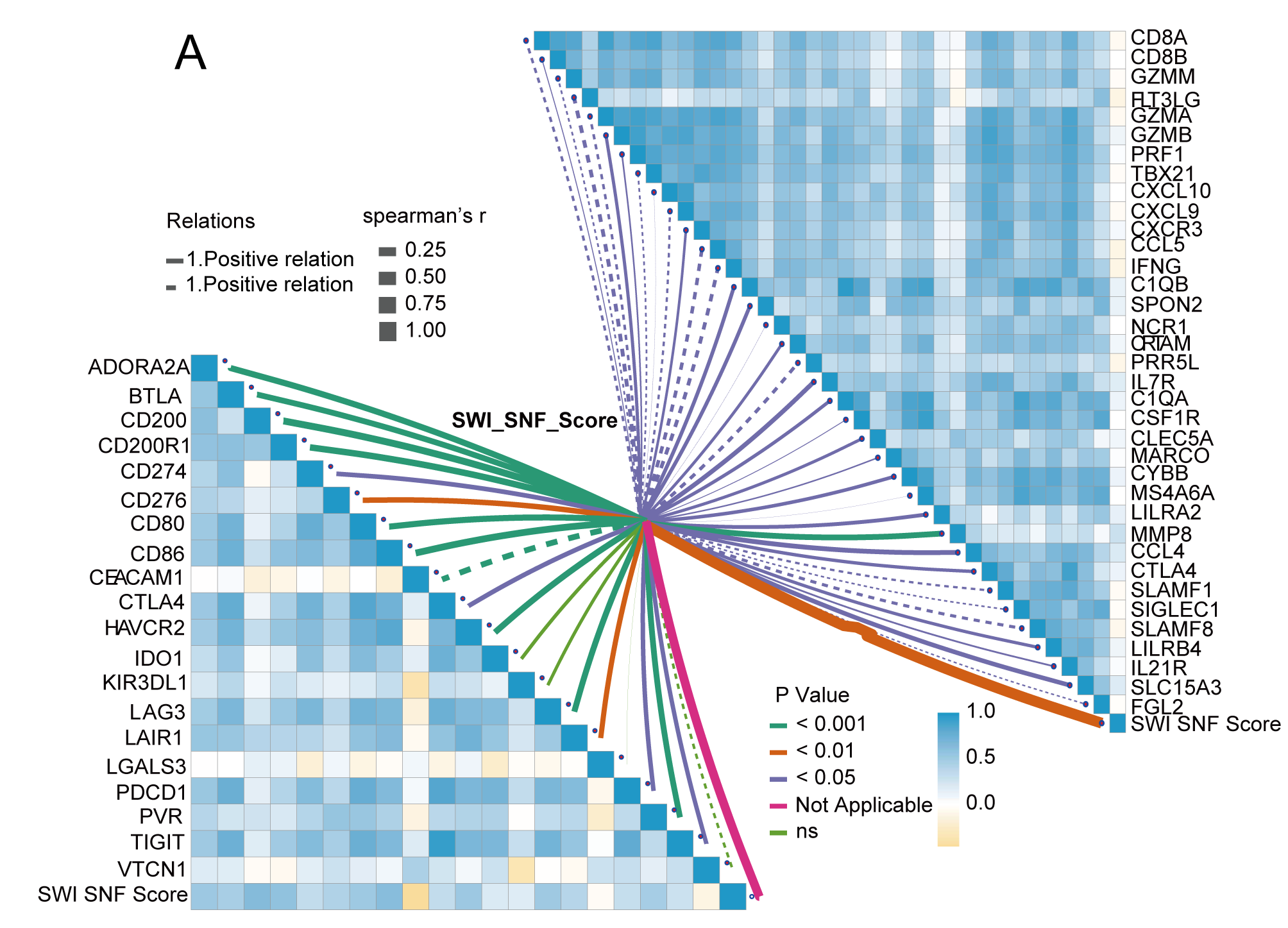

Supplement: Supplementary Figure 2 — (A) Correlations between the SWI_SNF_Score and immune checkpoint molecules, and correlations between the SWI_SNF_Score and immune cell characteristics. [file Image2.tif]

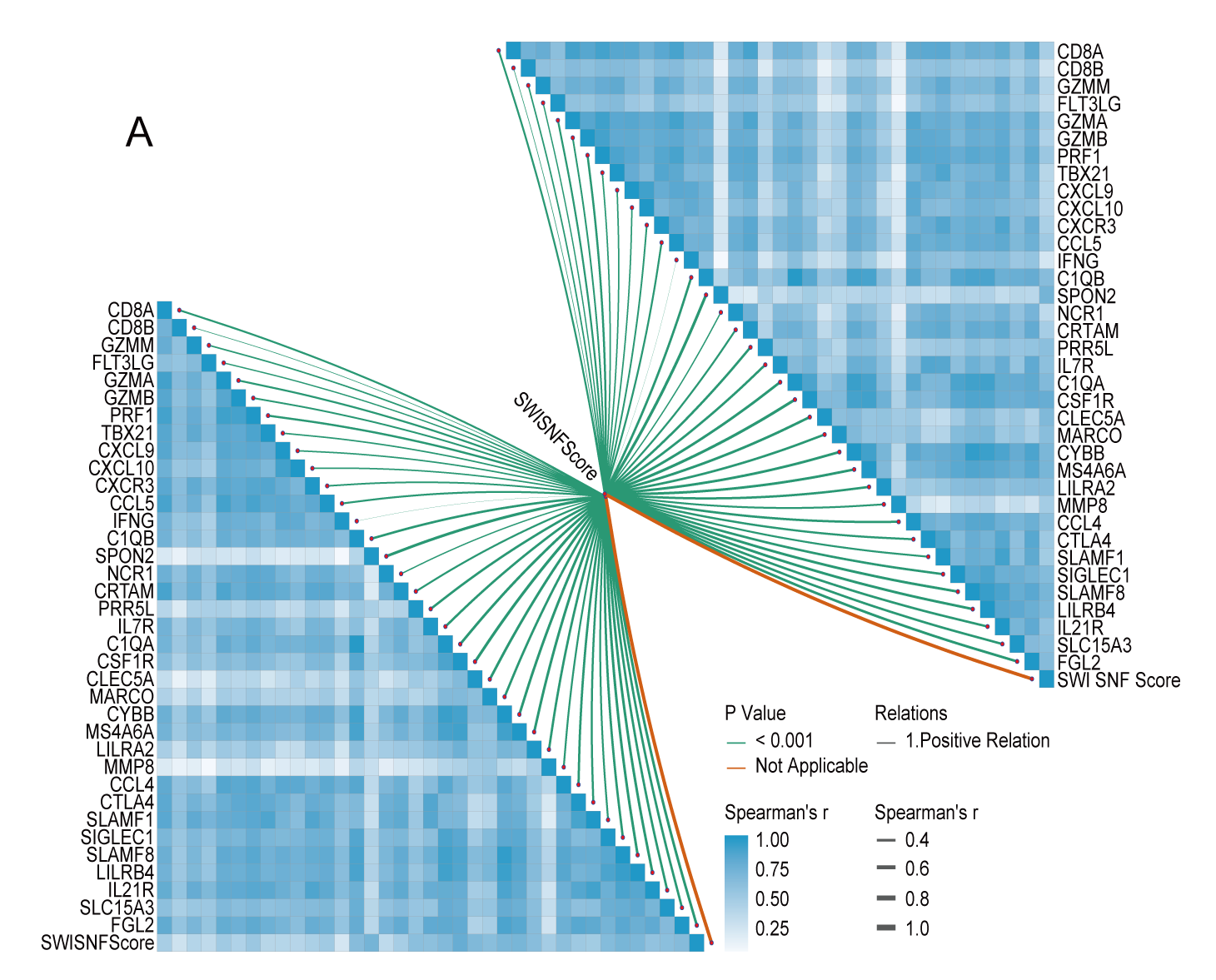

Supplement: Supplementary Figure 3 — (A) Correlation between the SWI_SNF_Score and effector genes of tumor-infiltrating lymphocytes (TILs). [file Image3.tif]
